# Preoperative Prediction Power of Radiomics for Breast Cancer: A Systemic Review and Meta-Analysis

**DOI:** 10.3389/fonc.2022.837257

**Published:** 2022-03-01

**Authors:** Zhenkai Li, Juan Ye, Hongdi Du, Ying Cao, Ying Wang, Desen Liu, Feng Zhu, Hailin Shen

**Affiliations:** ^1^ Department of Radiology, Suzhou Kowloon Hospital, Shanghai Jiaotong University School of Medicine, Suzhou, China; ^2^ Department of Radiotherapy, Suzhou Kowloon Hospital, Shanghai Jiaotong University School of Medicine, Suzhou, China; ^3^ Department of Thoracic Surgery, Suzhou Kowloon Hospital, Shanghai Jiaotong University School of Medicine, Suzhou, China

**Keywords:** breast cancer, radiomics, cancer prediction, meta-analysis, systematic review

## Abstract

**Background:**

To evaluate the preoperative predictive value of radiomics in the diagnosis of breast cancer (BC).

**Methods:**

By searching PubMed and Embase libraries, our study identified 19 eligible studies. We conducted a meta-analysis to assess the differential value in the preoperative assessment of BC using radiomics methods.

**Results:**

Nineteen radiomics studies focusing on the diagnostic efficacy of BC and involving 5865 patients were enrolled. The integrated sensitivity and specificity were 0.84 (95% CI: 0.80–0.87, *I* ^2 =^ 76.44%) and 0.83 (95% CI: 0.78–0.87, *I* ^2 =^ 81.79%), respectively. The AUC based on the SROC curve was 0.91, indicating a high diagnostic value.

**Conclusion:**

Radiomics has shown excellent diagnostic performance in the preoperative prediction of BC and is expected to be a promising method in clinical practice.

## Introduction

Breast cancer (BC) is the most commonly diagnosed cancer among women, accounting for 23% of all female cancers worldwide, and its related mortality is increasing by 4% each year (1). Traditional screening methods for BC, including X-ray mammography (MMG), breast ultrasound (US), and breast magnetic resonance imaging (MRI), rely mainly on qualitative characteristics, such as the lesion density, shape of lesion margins, and enhancement pattern. These imaging methods for BC screening have limitations in the sensitivity and specificity of diagnosis. As a result, biopsies are often performed to provide a definitive diagnosis for the patient. In the era of precision medicine, improvements in the performance of BC detection are urgently needed to reduce unnecessary biopsies, which are invasive and painful. Radiomics is an emerging application that can extract innumerable quantitative image features (including descriptors of tumor shape, size, intensity, and texture) that are difficult to recognize with the naked eye from almost any medical image (2). The traditional imaging diagnostic mode is more dependent on the experience of radiologists and has strong subjectivity. Compared with traditional imaging diagnosis mode, radiomics is an emerging application that can extract innumerable quantitative image features (including descriptors of tumor shape, size, intensity, and texture) that are difficult to recognize with the naked eye from almost any medical image (2). These image features may be related to the microscopic structure and tissue biological information of tumors. Based on this, combined with clinical, pathological and genetic information, the imaging support system for clinical decision making can be constructed. A number of studies have shown that the radiomics model can improve the accuracy of breast cancer diagnosis by extracting texture features of lesion and contralateral normal breast respectively and constructing benign/malignant classifiers (3, 4). Radiomics features have proven to be of significant value in differentiating between benign and malignant breast tumors (5, 6).Therefore, radiomics provides a promising method for improving the sensitivity and specificity of the diagnosis of BCs. By refining BC detection, radiomics has the potential to reduce unnecessary invasive biopsies. In addition, Shimauchi et al. (7) found that the performance of radiologists during diagnostic tasks improved when a computer-aided diagnosis system was used. Hence, the purpose of this study was to evaluate the diagnostic efficacy of radiomics in predicting BC.

## Materials and Methods

### Literature Retrieving

PubMed and EMBASE databases were comprehensively searched by two reviewers (L-ZK and YJ) using the following keywords: radiomics and BC, breast carcinoma, breast tumor, or breast neoplasm. The deadline of this retrieval was September 10, 2021. Two reviewers independently screened the abstracts of all manuscripts, and full publications were downloaded when the decision of including an article was ambiguous. Discussion was conducted to resolve disagreements on article inclusion. The reference lists of eligible studies were also searched for potential additional studies.

### Selection Criteria

The inclusion criteria were as follows: (1) diagnosis of BC on the basis of pathologic criteria; (2) breast imaging, including US, MRI, and/or digital MMG, was performed before biopsy or resection; and (3) radiomics analysis based on breast images was conducted.

The exclusion criteria were as follows: (1) preoperative administration of anticancer therapy (chemotherapy or radiotherapy); (2) the pathological diagnosis was not clear; and (3) imaging analysis based only on non-radiomics methods.

### Data Extraction and Study Quality Assessment

Two investigators (L-ZK and YJ) independently extracted the number of BC and non-BC cases, sensitivity, and specificity reported in the eligible studies. Using these data, we calculated the number of true positive (TP), false positive (FP), true negative (TN), and false negative (FN) results. If there were more than one model in the same group of patients, we used the model with the higher diagnostic accuracy in our meta-analysis. All studies included were quality assessed using the QUADAS-2 scale (8) in Revman 5.5 (Cochrane Library Software, Oxford, UK).

### Statistical Analysis

The pooled sensitivity and specificity were estimated. We also calculated pooled positive and negative likelihood ratios. Heterogeneity between the included studies was assessed by Cochrane’s Q-test and *I* ^2^ statistics. The summary receiver operating characteristic (SROC) curve and the area under the SROC curve (AUC) were also constructed to evaluate the diagnostic value of combined studies (9). AUCs of 0.5–0.7 indicated low diagnostic power, AUCs of 0.7–0.9 indicated moderate diagnostic power, and AUCs of 0.9–1.0 indicated high diagnostic power (10, 11). All statistical analyses were performed using Stata version 15.0 (Stata Corp), and *P*< 0.05 was considered statistically significant.

## Results

### Literature Selection and Quality Assessment

**Figure 1:**
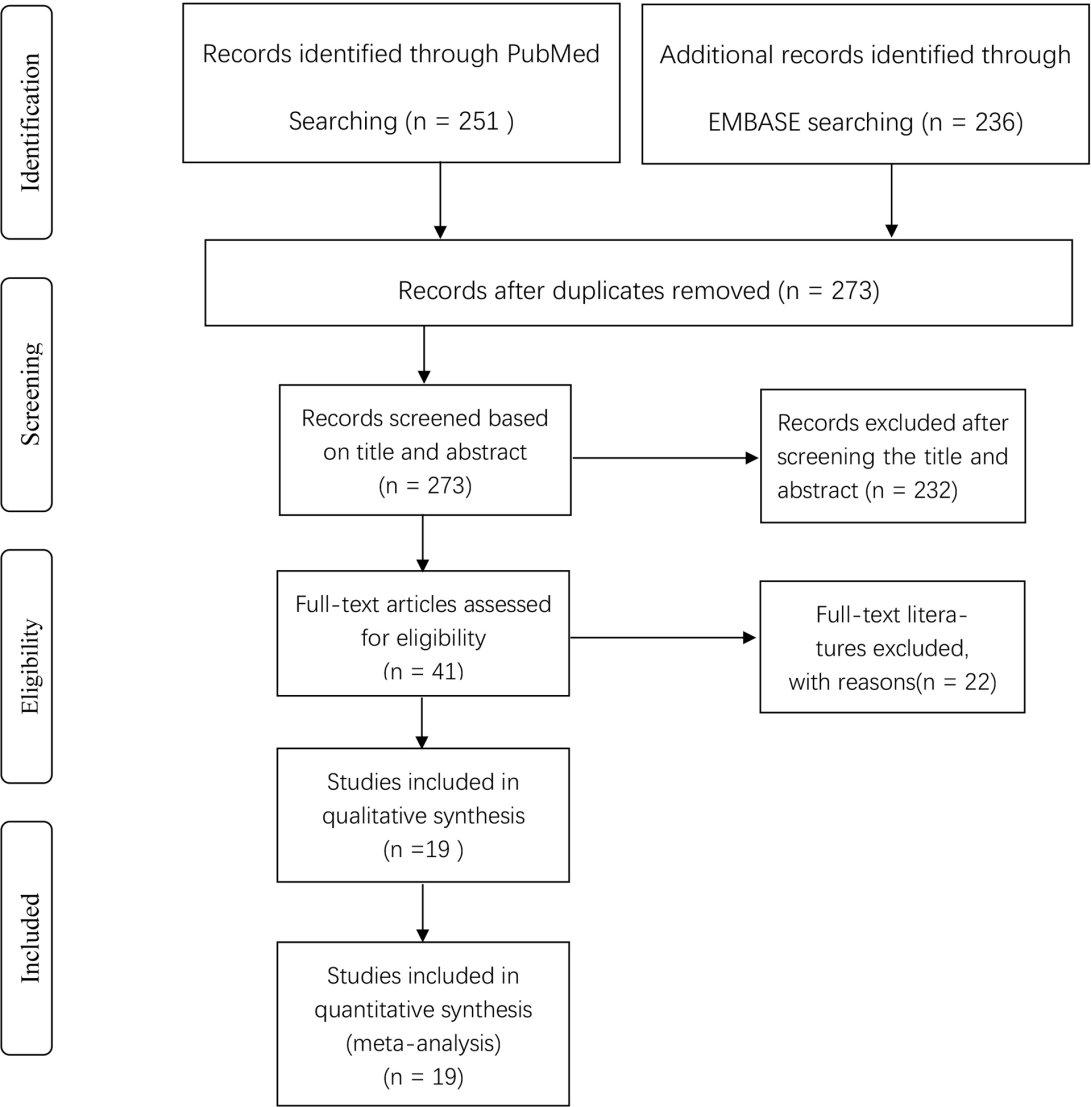
Flow diagram of literature screening according to PRISMA. PRISMA, Preferred Reported Items for Systematic Reviews and Metaanalyses.

**Figure 2:**
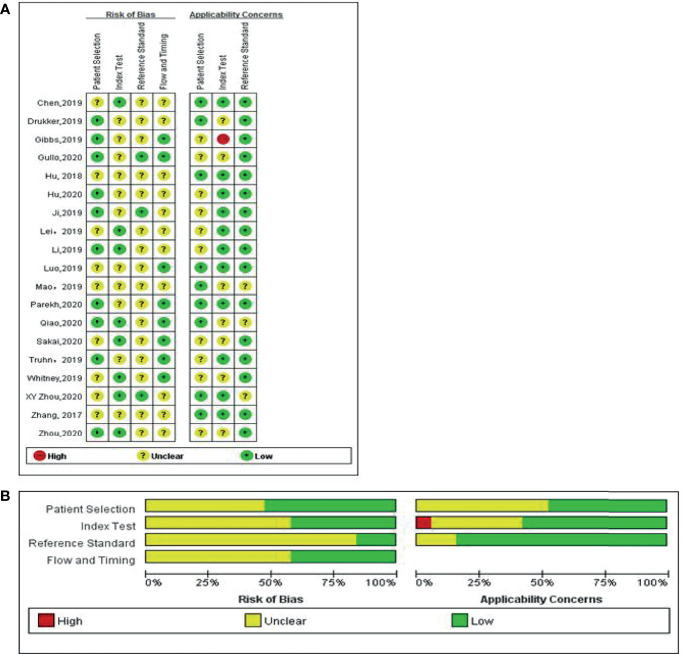
Methodological quality of the studies included in the meta-analysis according to the QUADAS 2 tool for risk of bias and applicability concerns. Green, yellow, and red circles represent low, unclear, and high risk of bias, respectively. **(A)** Individual studies, **(B)** summary.

Details of the selection procedure are shown in **Figure 1**. After the removal of duplicate articles, we reviewed the abstracts of 219 articles identified in the initial review. Nineteen eligible studies involving 5865 patients were included (12–30). Among them, 3500 patients were pathologically diagnosed with BC and 2365 patients as non-BC. The basic characteristics of all eligible studies are displayed in **Table 1**, and the quality assessment of all included studies based on the QUADAS-2 scale is shown in **Figure 2**.

**Table 1:** Basic characteristics.

Study	Study Design	Region	NO.	Radiomics algorithm	Subgroup	Imaging modality	BC	non-BC	TP	FP	FN	TN	Feature type
Zhang,2017 (28)	Retrospective	China	117	Conventional algorithm	Conventional image	US	42	75	36	8	6	67	7 radiomic features
Hu,2018 (15)	Retrospective	China	88	Machine learning	Functional image	MRI	52	36	42	8	10	28	5 radiomic features
Luo,2019 (21)	Retrospective	China	315	Conventional algorithm	Conventional image	US	68	143	52	24	16	119	9 radiomic features
							35	69	29	5	6	64	
Li,2019 (19)	Retrospective	USA	182	Conventional algorithm	Conventional image	MMG	106	76	83	18	23	58	32 radiomic features
Drukker,2019 (13)	Prospective	USA	109	Conventional algorithm	Conventional image	MMG	35	74	34	36	1	38	9 radiomic features
Whitney,2019 (27)	Retrospective	USA	462	Deep learning	Functional image	MRI	296	212	222	46	74	166	38 radiomic features
Ji,2019 (17)	Retrospective	China	1979	Machine learning	Functional image	MRI	421	114	352	20	69	94	10 radiomic features
Gibbs,2019 (14)	Retrospective	USA	149	Conventional algorithm	Functional image	MRI	9	32	6	0	3	32	4 Clinical and 1 radiomics feature
Chen,2019 (12)	Retrospective	China	81	Conventional algorithm	Functional image	MMG, MRI	40	41	33	8	7	33	14 radiomic features
Truhn, 2019 (26)	Retrospective	Germany	447	Conventional algorithm	Functional image	MRI	787	507	616	78	171	429	10 radiomic features
Lei, 2019 (18)	Retrospective	China	419	Conventional algorithm	Conventional image	MMG	78	81	63	18	15	63	6 radiomic features
							28	25	25	9	3	16	
Mao, 2019 (22)	Retrospective	China	173	Conventional algorithm	Conventional image	MMG	79	59	78	1	1	58	51 radiomic features
							20	15	17	1	3	14	
Gullo,2020 (20)	Retrospective	USA	430	Conventional algorithm	Functional image	MRI	40	76	25	7	15	69	1 Clinical and 10 radiomics features
Hu,2020 (16)	Retrospective	USA	612	Conventional algorithm	Functional image	MRI	657	159	520	36	137	123	75 radiomic features
Parekh,2020 (23)	Retrospective	USA	138	Conventional algorithm	Functional image	MRI	97	41	80	8	17	33	10 radiomic features
Qiao,2020 (24)	Retrospective	China	267	Conventional algorithm	Functional image	MRI	136	131	115	25	21	106	246 radiomic features
XY Zhou,2020 (30)	Retrospective	China	228	Conventional algorithm	Functional image	MRI	158	70	149	5	9	65	9 radiomic features
Zhou,2020 (29)	Retrospective	China	227	Deep learning	Functional image	MRI	91	62	83	17	8	45	1 Clinical and 5 radiomics features
							48	26	41	9	7	17	
Sakai,2020 (25)	Retrospective	Japan+USA	24	Machine learning	Conventional image	MMG	31	20	21	5	10	15	6 radiomic features

MMG, Mammography; US, Ultrasound; MRI, Magnetic Resonance Imaging; BC, Breast cancer; TP, True positive; FP, False positive; TN, True negative; FN, False negative.

### Radiomics for the Preoperative Prediction of BC

**Figure 3:**
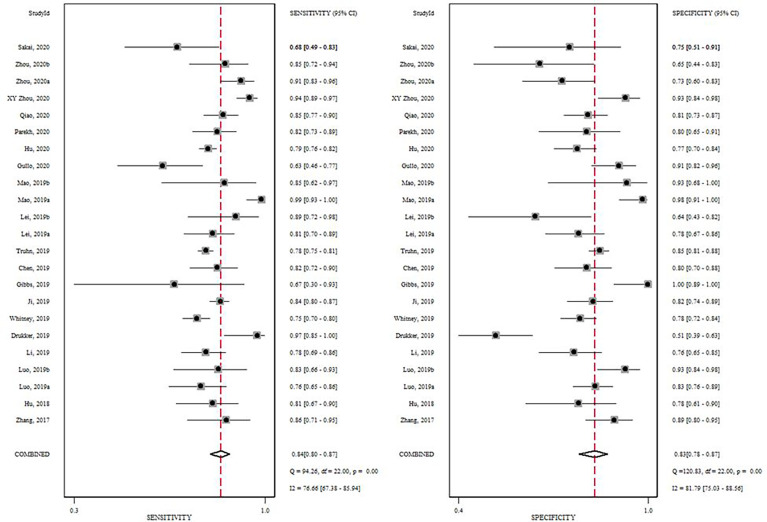
Forrest plot of the effect size calculated as log odds ratio for 19 studies investigating the diagnostic accuracy of radiomics in the differentiation of BC from breast masses. Numbers are pooled estimates, with 95% confidence intervals (CIs) depicted with horizontal lines. Heterogeneity statistics are shown at bottom right.

**Figure 4:**
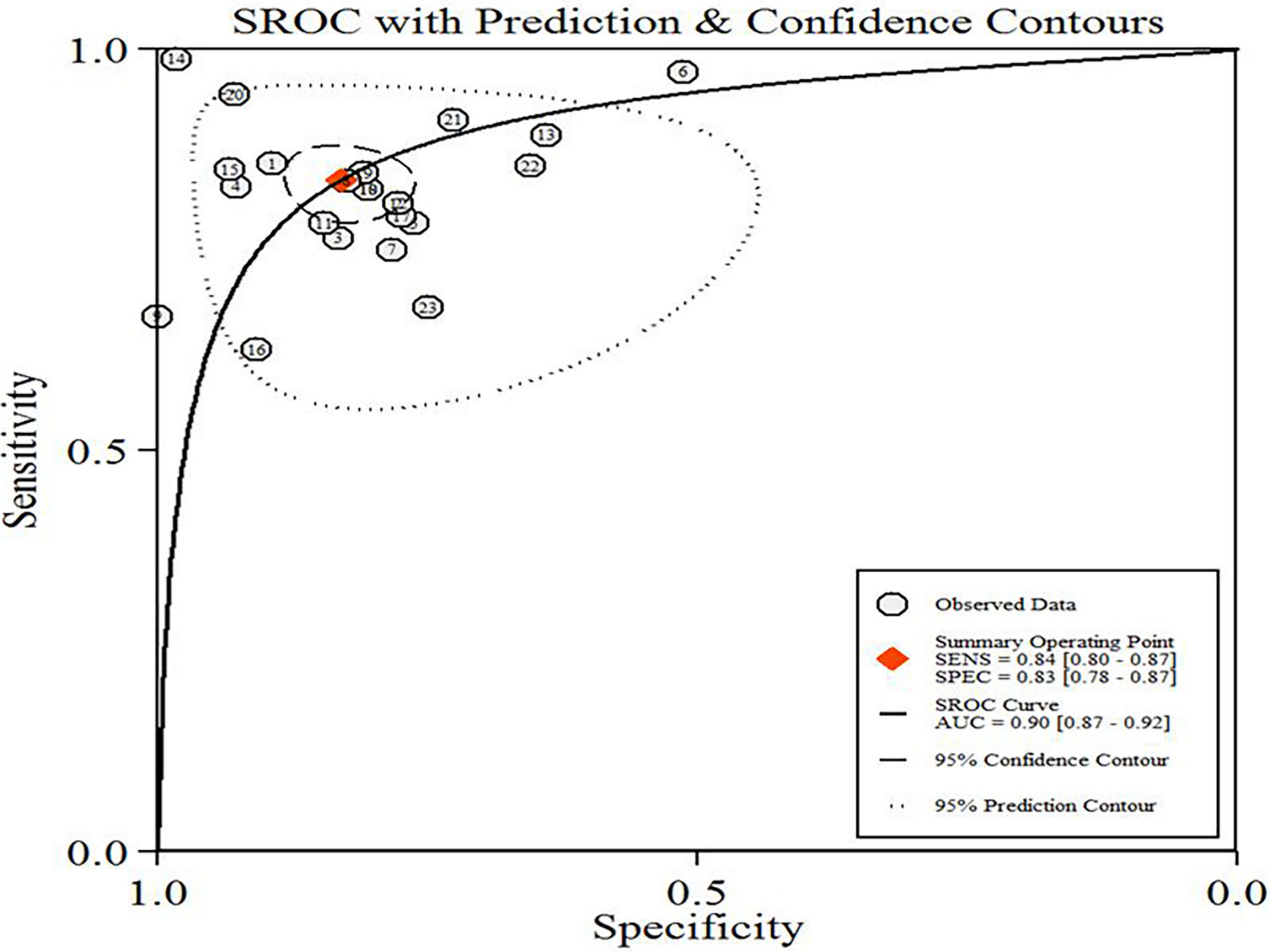
Hierarchical summary receiver operating characteristic curve (SROC) plot of diagnostic performance in predicting BC of the included radiomic models. The numbers in circles correspond to the order of the articles in **Table 1**.

A total of 5865 patients, comprising 3500 BC and 2365 non-BC patients, were assessed using a radiomics method. **Figure 3** shows the forest plots of the diagnostic meta-analysis and combined results. The integrated sensitivity and specificity were 0.84 (95% CI: 0.80–0.87, *I* ^2^ = 76.44%) and 0.83 (95% CI: 0.78–0.87, *I* ^2^ = 81.79%), respectively. The AUC based on the SROC curve was 0.91 (**Figure 4**), demonstrating a high diagnostic value.

### Subgroup Analyses and Sensitivity Analyses

Subgroup analyses were performed and included five different conditions and eleven subgroups. Radiomics models showed moderate to high diagnostic value in each subgroup of imaging modalities (MMG, US, and MRI), study design (prospective and retrospective), data source (China and America), modeling method [Radiomics algorithm (RA), machine learning (ML), and deep learning (DL)]. Both conventional and functional imaging analyses provided a high diagnostic accuracy of BC. The results are displayed in **Table 2**. Repeating the meta-analyses after removing studies of adjusted unreported variables did not change our findings (**Table 3**).

**Table 2:** Subgroup analyses.

Subgroup	Number of study	Sensitivity	Specificity	PLR	NLR	AUC
**Main effect**	19	0.84	0.83	4.9	0.2	0.9
**Imaging modality**						
MRI	9	0.83	0.82	4.5	0.21	0.89
MMG	6	0.87	0.79	4.2	0.16	0.91
US	5	0.82	0.87	6.2	0.21	0.9
**Study Design**						
Prospective	1	0.97	0.51	2.2	0.44	0.84
Retrospective	18	0.83	0.84	5.2	0.2	0.9
**Region**						
China	10	0.86	0.85	5.7	0.16	0.92
USA	8	0.77	0.8	3.9	0.28	0.85
**Imaging technique**						
Conventional image	6	0.88	0.82	4.9	0.15	0.92
Functional image	13	0.82	0.83	4.9	0.22	0.9
**Modeling methods**						
Radiomic algorithm	5	0.87	0.8	4.3	0.16	0.91
Machine/Deep learning	14	0.83	0.84	5.2	0.21	0.9

MMG, Mammography; US, Ultrasound; MRI, Magnetic Resonance Imaging; AUC, Area under curve; PLR, Positive likelihood ratio; NLR, Negative likelihood ratio.

**Table 3:** Sensitivity analyses.

Study removed	Sensitivity	Specificity	PLR	NLR	AUC
No	0.84	0.83	4.9	0.2	0.9
Zhang, 2017 (28)	0.84	0.83	4.8	0.2	0.9
Hu, 2018 (15)	0.84	0.83	5	0.19	0.9
Luo, 2019 (21)	0.84	0.82	4.8	0.19	0.9
Li, 2019 (19)	0.84	0.83	5	0.19	0.9
Drukker, 2019 (13)	0.83	0.84	5.2	0.2	0.9
Whitney, 2019 (27)	0.84	0.83	5	0.19	0.9
Ji, 2019 (17)	0.84	0.83	5	0.2	0.9
Gibbs, 2019 (14)	0.84	0.82	4.7	0.2	0.9
Chen, 2019 (12)	0.84	0.83	5	0.19	0.9
Truhn, 2019 (26)	0.84	0.83	4.9	0.19	0.9
Lei, 2019 (18)	0.84	0.84	5.2	0.19	0.91
Mao, 2019 (22)	0.82	0.81	4.4	0.22	0.89
Gullo, 2020 (20)	0.84	0.84	4.9	0.19	0.9
Hu, 2020 (16)	0.84	0.83	5	0.19	0.9
Parekh, 2020 (23)	0.84	0.83	5	0.19	0.9
Qiao, 2020 (24)	0.84	0.83	5	0.2	0.9
XY Zhou, 2020 (30)	0.83	0.82	4.7	0.21	0.89
Zhou, 2020 (29)	0.84	0.83	5.2	0.2	0.9
Sakai, 2020 (25)	0.84	0.83	5	0.19	0.9

AUC, Area under curve; PLR, Positive likelihood ratio; NLR, Negative likelihood ratio.

## Discussion

We compared the preoperative predictive value of radiomics in the diagnostic performance of BC in different studies. The results showed that the diagnostic value of radiomics was high in predicting BC with an aggregated sensitivity, specificity, and AUC of 0.84, 0.83, and 0.91, respectively. Although the number and types of features varied among the 19 included studies, which may influence the aggregated sensitivity and specificity, radiomics was shown to have good predictive ability of BC in each study. Significant heterogeneity was identified in our study. Specifically, the screening methods, selection of the scanner manufacturer and model, acquisition methods, and reconstruction parameters were shown to contribute to the heterogeneity in imaging data.

Sensitivity analyses showed that our results were reliable and stable after each study was sequentially removed, and the unreported adjusted variables were omitted.

The specificity and AUC of conventional and functional imaging analyses were similar, but conventional imaging had a slightly higher sensitivity. A possible explanation is that conventional imaging analyses included in this meta-analysis were combined with special examination methods. Li et al. combined the radiomic analysis of breast tumors and the parenchyma to improve the diagnostic accuracy of BC (19). Luo et al. used a nomogram combined with radiomics and the Breast Imaging Reporting and Data System (BI-RADS) score to predict BC (21). As for imaging modalities, the sensitivity and AUC of predicting BC by MMG were slightly higher than those of MRI and US, but the specificity was slightly lower. The use of new ultrasound imaging techniques, such as ultrasound elastography and contrast-enhanced ultrasound, may improve the detection of BC. Multi-modal MRI imaging techniques can detect most early-stage BCs, and the specificity of MRI is usually higher than that of MMG and US (31). In terms of modeling methods, ML and DL were widely studied, among which support vector machines and convolutional neural networks were the most commonly used. Logistic regression was also applied because the status of the breast mass (BC or non-BC) is a dichotomous variable. The results showed that radiomics based on either modeling method could achieve high diagnostic efficiency in predicting BC. Lastly, different data sources and study designs influenced the aggregated sensitivity, specificity, and AUC. Thus, more studies focusing on these subgroups are needed.

The preoperative diagnosis and clinical staging of BC are implemented mainly through the visual observation and analysis of medical images. The BI-RADS (32) score is a standardized description of imaging features of breast tumors, and it provides an approximate risk of malignancy to a lesion but lacks a characteristic evaluation of the intrinsic heterogeneity in tumors reflecting different biological behaviors of BC. To overcome limitations in the observation of tumor images by the naked eye, artificial intelligence has been increasingly applied to the mining and use of medical image data to meet the growing need for individualized evaluation (33). With the in-depth study of radiomics, models based on radiomics features have been shown to be a promising non-invasive method for BC classification and prediction (34). Reportedly, radiomics models based on features extracted from preoperative MMG, US, or MRI images had a relatively high predictive performance (12–30). Texture feature analysis based on US sonoelastography was first used to propose a quantitative radiomics approach for the feature selection and classification of breast tumors (28). Subsequently, many studies performed feature extraction from multi-parameter MRI images, including T2-weighted (T2w) MRI sequences, diffusion-weighted imaging (DWI) sequences, and dynamic contrast-enhanced (DCE)-MRI sequences, and constructed well-performed radiomics predictive models using ML or DL methods (12, 15, 16, 20, 23, 24, 26, 29, 30). A recent study suggested that mammography radiomics combined with quantitative three-compartment breast image analysis could reduce unnecessary breast biopsies (13).

Our meta-analysis of preoperative BC prediction using radiomics methods has two advantages. First, to the best of our knowledge, this study involving 19 articles and 5865 breast masses is the first meta-analysis to assess the diagnostic efficacy of radiomics models in predicting BC before surgery. Secondly, this study evaluated the diagnostic efficacy of radiomics models in predicting BC by comparing imaging modalities, modeling methods, and other subgroups, thereby providing ideas for subsequent radiomics research.

There are several inherent limitations to this study that need to be discussed. First, the methodology of radiomics studies included in this analysis was different as different medical centers use various examination equipment, and the selection of imaging modality, feature extraction, and modeling methods provides an infinite number of combinations. Second, the code used for feature extraction and model building was not publicly available for any of the 19 studies included in this analysis, preventing replication and independent validation of the research results. Third, because our study used summary statistics rather than individual raw data, it was not possible to achieve more reliable results. However, it was possible to achieve more precise delineation and control potential residual confounding, a common limitation of meta-analyses.

## Conclusions

Our study shows that radiomics models based on preoperative imaging features are useful for the prediction of BC and have high diagnostic efficacy and consistency among studies. Radiomics is expected to provide a new quantitative diagnostic method for clinical work, but more well-designed prospective radiomics trials are needed to demonstrate its effectiveness and ability to translate into clinical practice.

## Data Availability Statement

The original contributions presented in the study are included in the article/supplementary material. Further inquiries can be directed to the corresponding author.

## Author Contributions

Guarantor of the integrity of the study: HS. Study concepts: JY. Literature research: ZL. Data acquisition: HD and YW. Data analysis: DL and ZL. Statistical analysis: HS and FZ. Manuscript preparation: ZL and YC. Manuscript editing: ZL and YC. Manuscript review: DL, HS, and JY.

## Conflict of Interest

The authors declare that the research was conducted in the absence of any commercial or financial relationships that could be construed as a potential conflict of interest.

## Publisher’s Note

All claims expressed in this article are solely those of the authors and do not necessarily represent those of their affiliated organizations, or those of the publisher, the editors and the reviewers. Any product that may be evaluated in this article, or claim that may be made by its manufacturer, is not guaranteed or endorsed by the publisher.

## Abbreviations

BC, Breast cancer; CI, Confidence interval; ROC, Receiver operating characteristic; SROC, Summary receiver operating characteristic; AUC, Area under curve; MMG, Mammography; US, Ultrasound; MRI, Magnetic Resonance Imaging; TP, True positive; FP, False positive; TN, True negative; FN, False negative; RA, Radiomics algorithm; ML, Machine learning; DL, Deep learning; BI-RADS, Breast Imaging Reporting and Data System; DWI, Diffusion-weighted imaging; DCE, Dynamic contrast-enhanced.
